# Counting every stillbirth and neonatal death through mortality audit to improve quality of care for every pregnant woman and her baby

**DOI:** 10.1186/1471-2393-15-S2-S9

**Published:** 2015-09-11

**Authors:** Kate J Kerber, Matthews Mathai, Gwyneth Lewis, Vicki Flenady, Jan Jaap HM Erwich, Tunde Segun, Patrick Aliganyira, Ali Abdelmegeid, Emma Allanson, Nathalie Roos, Natasha Rhoda, Joy E Lawn, Robert Pattinson

**Affiliations:** 1 Saving Newborn Lives, Save the Children, 2000 L Street NW, Suite 500, Washington, DC 20036, USA; 2 Department of Maternal, Newborn, Child and Adolescent Health, World Health Organization, 20 Avenue Appia, 1211 Geneva 27, Switzerland; 3 Institute for Women's Health, University College London, 74 Huntley Street, London WC1E 6AU, United Kingdom; 4 Translating Research Into Practice Centre, Mater Research Institute, University of Queensland, Aubigny Place, South Brisbane, Qld 4101, Australia; 5 Department of Obstetrics and Gynecology, University Medical Center Groningen, University of Groningen, Homepostcode CB20, PO Box 30 001, 9700 RB Groningen, The Netherlands; 6 Evidence for Action, 19B Jimmy Carter Street, Asokoro, Abuja, Nigeria; 7 Save the Children, Plot 68/70 Kira Road, Kampala, Uganda; 8 JHPIEGO, 1776 Massachusetts Ave., NW, Washington, DC 20036, USA; 9 School of Women's and Infants' Health, Faculty of Medicine, Dentistry and Health Sciences, University of Western Australia, 35 Stirling Highway, Crawley, 6009, Australia; 10 UNDP/UNFPA/UNICEF/WHO/World Bank Special Programme of Research, Development and Research Training in Human Reproduction (HRP), Department of Reproductive Health and Research, World Health Organization, Avenue Appia 20, Geneva, CH-1211, Switzerland; 11 University of Cape Town, Groote Schuur Hospital, Main Road, Observatory, 7925, South Africa; 12 Maternal, Adolescent, Reproductive and Child Health (MARCH) Centre, London School of Hygiene and Tropical Medicine, London, WC1E 7HT, UK; 13 Department of Infectious Disease Epidemiology, London School of Hygiene and Tropical Medicine, London, WC1E 7HT, UK; 14 SAMRC Maternal and Infant Health Care Strategies Unit, Obstetrics and Gynaecology Department, University of Pretoria, PO Box 323 Arcardia, 0007, South Africa

**Keywords:** Healthworker, medical audit, maternal, midwives, mortality, neonatal death, stillbirth, quality of care

## Abstract

**Background:**

While there is widespread acknowledgment of the need for improved quality and quantity of information on births and deaths, there has been less movement towards systematically capturing and reviewing the causes and avoidable factors linked to deaths, in order to affect change. This is particularly true for stillbirths and neonatal deaths which can fall between different health care providers and departments. Maternal and perinatal mortality audit applies to two of the five objectives in the Every Newborn Action Plan but data on successful approaches to overcome bottlenecks to scaling up audit are lacking.

**Methods:**

We reviewed the current evidence for facility-based perinatal mortality audit with a focus on low- and middle-income countries and assessed the status of mortality audit policy and implementation. Based on challenges identified in the literature, key challenges to completing the audit cycle and affecting change were identified across the WHO health system building blocks, along with solutions, in order to inform the process of scaling up this strategy with attention to quality.

**Results:**

Maternal death surveillance and review is moving rapidly with many countries enacting and implementing policies and with accountability beyond the single facility conducting the audits. While 51 priority countries report having a policy on maternal death notification in 2014, only 17 countries have a policy for reporting and reviewing stillbirths and neonatal deaths. The existing evidence demonstrates the potential for audit to improve birth outcomes, only if the audit cycle is completed. The primary challenges within the health system building blocks are in the area of leadership and health information. Examples of successful implementation exist from high income countries and select low- and middle-income countries provide valuable learning, especially on the need for leadership for effective audit systems and on the development and the use of clear guidelines and protocols in order to ensure that the audit cycle is completed.

**Conclusions:**

Health workers have the power to change health care routines in daily practice, but this must be accompanied by concrete inputs at every level of the health system. The system requires data systems including consistent cause of death classification and use of best practice guidelines to monitor performance, as well as leaders to champion the process, especially to ensure a no-blame environment, and to access change agents at other levels to address larger, systemic challenges.

## Background

Access to reliable data detailing the numbers and causes of death within Civil Registration and Vital Statistics (CRVS) and beyond is essential for programme planning and monitoring. Surveillance and response for maternal deaths is becoming an increasingly popular strategy in high and low-income settings to collect accurate information linked to routine health systems on how many women died, where they died, why they died, and what could have been done differently in order to prevent future similar deaths. The process promotes routine identification and timely notification of deaths and is a continuous action cycle linking quality improvement from local to national level [1]. Audit and feedback shows a greater impact on health care practices and outcomes than other quality improvement strategies, particularly in settings where there is greater opportunity for improvement, and when the audit process includes an action plan and clear targets [2].

Mortality audit for maternal deaths, which focuses primarily on using data and peer review to improve quality of care, has a long history [3]. This has recently been expanded in some settings to include maternal death surveillance, which has the additional elements of systematic collection and analysis of every death at all levels of the health system [1]. Both include widespread acknowledgment of the need for better information on births and deaths and the need to interpret and act on that information. Despite the fact that women and their babies share the same period of highest risk, often with the same health workers present, there has been less movement towards capturing similar information for perinatal deaths. Each year, half of the world's babies do not receive a birth certificate; most neonatal deaths and almost all stillbirths have no death certificate, let alone information on the causes and avoidable factors surrounding these deaths [4].

**Figure 1:**
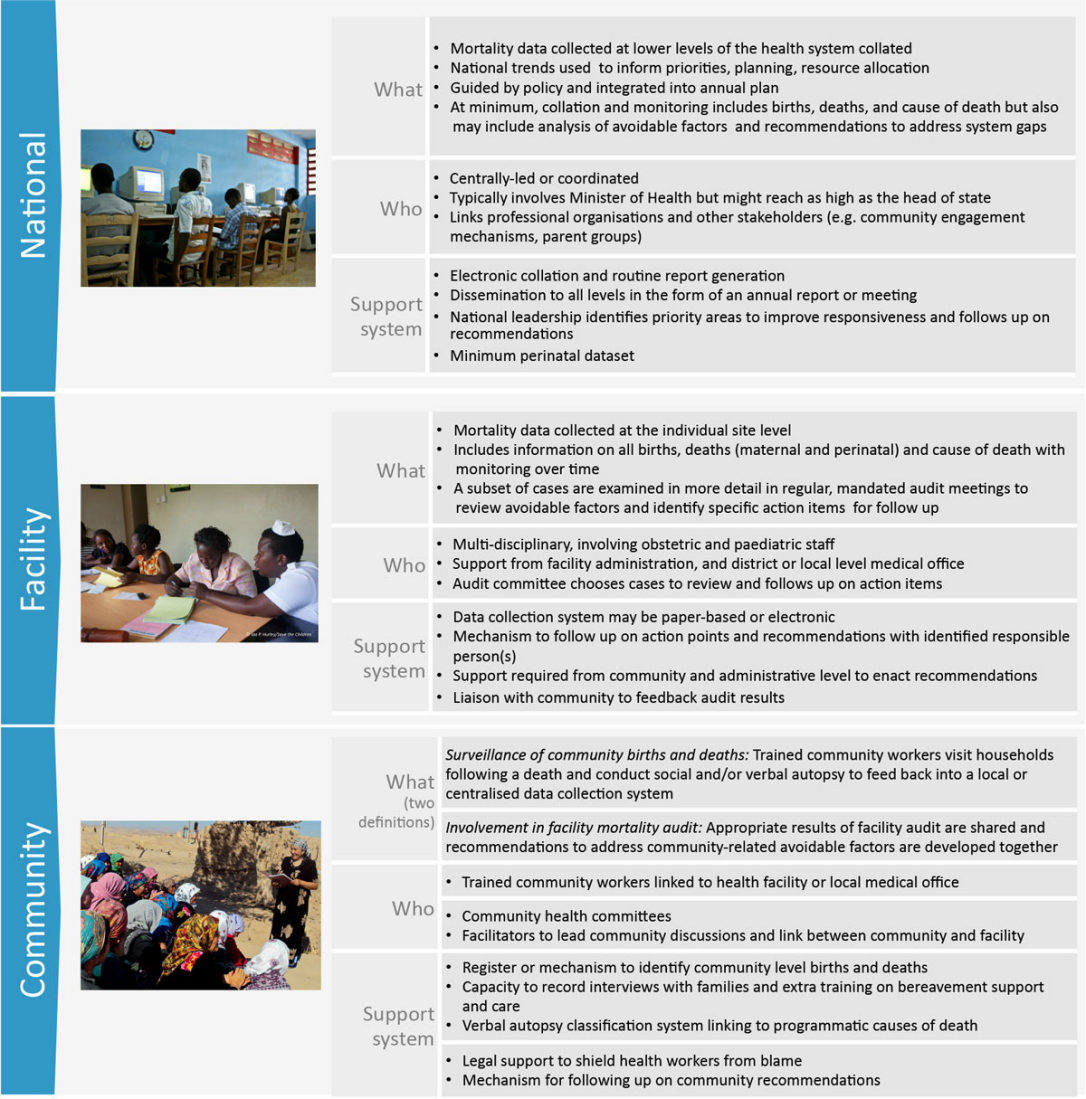
**Perinatal audit parameters by level of care**. National level image source: Save the Children. Facility level image source: Ian Hurley/Save the Children. Community level: Save the Children

Based on the description of Dunn and McIlwaine [5] and Crombie [6], we define perinatal outcome audit as the process of capturing information on the number and causes of all stillbirths and neonatal deaths, or near-misses where applicable, with an aim towards identifying specific cases for systematic, critical analysis of the quality of perinatal care received in a no-blame, interdisciplinary setting in order to improve the care provided to all mothers and babies. Mortality audit can have multiple entry points into the health system, ranging from a single hospital to a nationally-mandated programme covering community and facility level (Figure 1). Maternal and perinatal mortality audit is covered under two of the five objectives in the *Every Newborn *Action Plan: to address quality of care at birth and to generate data for decision making and action [7]. This is the ninth paper in the Every Woman, Every Newborn series on quality of care across the continuum of care. Other interventions explored in this series were subject to a stakeholder consultation process in 12 countries to identify health system bottlenecks, common themes and solutions to address gaps in providing quality care to mothers and newborns. Each of the countries listed perinatal mortality audit as a proposed solution for improving quality of care [8]. As one cross-cutting entry point which will act upon multiple interventions and approaches to help fill some of the gaps identified during country consultations, mortality audit was considered separately with the aim of describing the current evidence for mortality audit, assessing progress in policy uptake, and qualitatively identifying approaches to overcome challenges and scale up mortality audit for stillbirths and neonatal deaths.

The objectives of this paper are:

1. To review national policies and existing national and local systems to assess country progress towards institutionalising facility-based maternal and perinatal death audit

2. To review the available evidence for perinatal mortality audit and to synthesise the main challenges from the literature within the WHO health system building blocks

3. To propose solutions for scaling up mortality audit for stillbirths and neonatal deaths based on literature and programme learning.

## Methods

In order to track policy progress for mortality audit overall, we assessed the status of maternal death notification in Countdown to 2015 for Maternal, Newborn and Child Health [9] priority countries since tracking began in 2008. We also collected and reviewed policy and strategy documents and national guidelines through database searches and key informant inquiries in these priority countries to determine whether a process for perinatal mortality audit implementation was in place or underway at national level. We also reviewed the current evidence for facility-based perinatal mortality audit with a focus on low- and middle-income countries where the majority of the world's births and deaths occur.

Challenges to introducing, sustaining and achieving impact with perinatal mortality audit were identified in published and grey literature and programme learning documentation. Given the limited published information about perinatal mortality audit, lessons learned from maternal audit was also considered. Challenges and context-specific solutions were identified and categorised into thematic areas and linked to the WHO health system building blocks framework, adding the additional build block of community ownership and participation [10]. We undertook a literature review to identify further case studies and evidence-based solutions for each defined thematic area.

## Results

### Country progress for maternal and perinatal death audit

**Figure 2:**
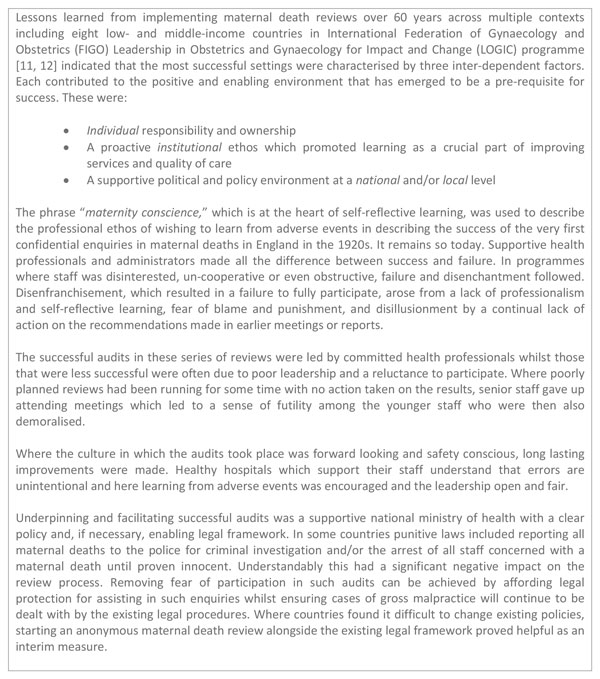
**Lessons learned from 60 years of confidential enquiries and maternal death review**. FIGO: International Federation of Gynecology and Obstetrics.

Maternal mortality audit has become more widespread and successful in many countries and has moved forward much more quickly than perinatal audit. The recent WHO Maternal Death Surveillance and Response (MDSR) technical guide [1] outlines the continuous action cycle that builds on established maternal death review processes. Such reviews, when carried out well, have led to local policy change and improvements in the quality of maternal health services, even in challenging settings (Figure 2) [11,12]. In addition to continuous surveillance of all maternal deaths and notification linked to the health information system and higher level policy actors, the MDSR approach also mandates that each death receives a systematic review and recommendations with actions to prevent similar deaths in the future [1].

**Figure 3:**
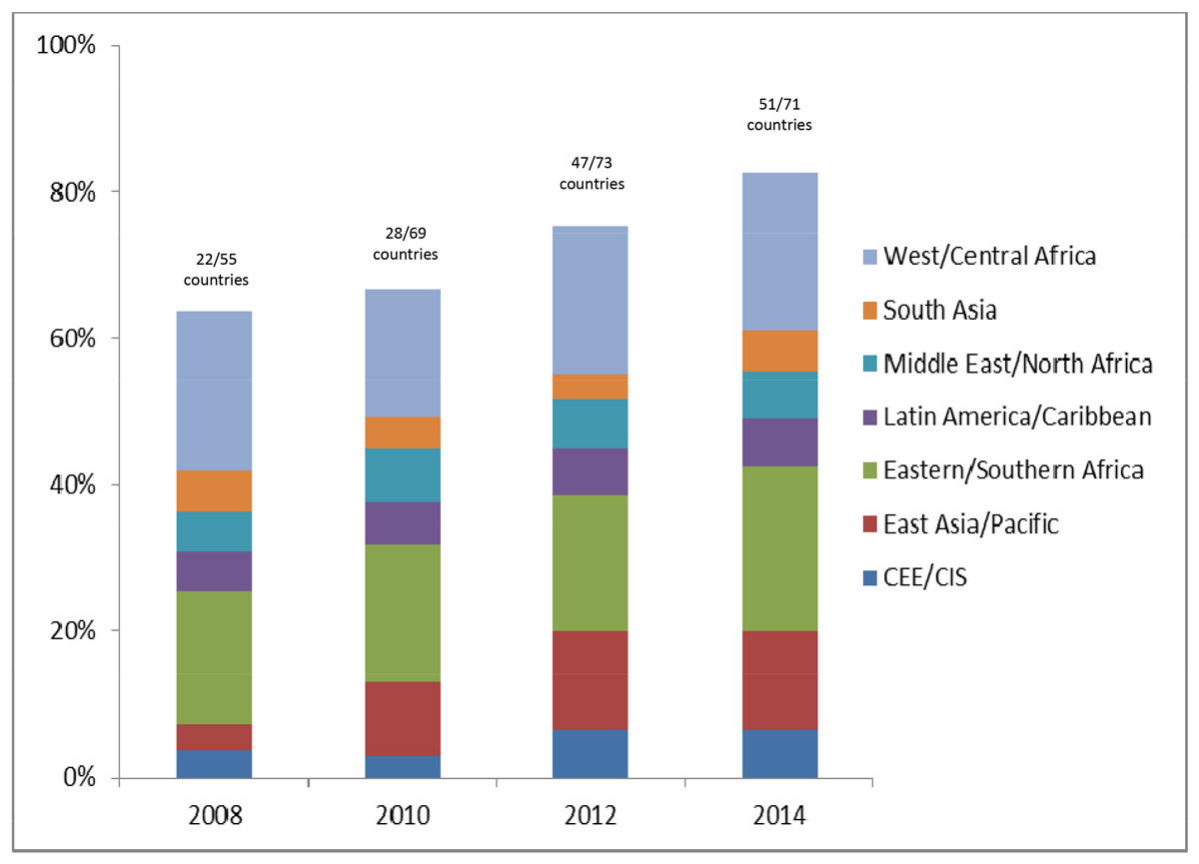
**Adoption of maternal and perinatal death notification in Countdown priority countries**. Countries with perinatal mortality audit in policy or a national system for facility review of **perinatal **deaths in 2014: 17 (Angola, Azerbaijan, Bangladesh, Gabon, Gambia, Indonesia, Iraq*, Kenya, Liberia*, Mexico*, South Africa, Rwanda, Tanzania, Uganda, Uzbekistan, Zambia*, Zimbabwe). *refers to stillbirths only or early neonatal only; not both. Sources: [9,13,61,62] See additional file 1 for more details.

Maternal deaths were notifiable by national policy in 51 of 71 (72%) high burden countries in 2014 [13], up from 22 of 55 countries (40%) in 2008 [14] (Figure 3) with more countries moving towards implementation in addition to policy (See Table S1, additional file 1). Only Haiti of the six Latin American and Caribbean countries did not have a policy by 2014. In 2014, all Countdown countries in the Central and Eastern Europe and the Commonwealth of Independent States (CEE/CIS) region (n = 5) had a documented policy for maternal death notification. The African Union call to make maternal deaths notifiable and institute maternal death reviews in all countries through the Campaign for Accelerated Reduction of Maternal Mortality in Africa (CARMMA) [15] may have had an impact on the continent, with only 36% of assessed countries having a maternal death notification policy in 2008 increasing to 70% of countries in 2014.

Perinatal death and maternal death audits can be performed by the same team using similar processes. For example, perinatal deaths could be discussed at the same review meetings as maternal deaths [16]. There was no evidence of a country having a policy for perinatal mortality audit without also having one in place for maternal mortality audit. Out of the priority countries, including the 51 which reported maternal death notification in policy in 2014, only 17 have a national mandate for perinatal death reviews (Figure 3 with more detail in Table S2, additional file 1). Evidence suggests that policy is a necessary condition for commencing implementation of audit processes, but policy alone is not sufficient for the running of an effective audit programme. While most countries have individual facilities (both public and private) already conducting perinatal mortality audits, few have systems in place that link the data to national level databases with accountability structures in place for the recommendations identified through the audit process. In Tanzania, for example, despite staff commitment to capturing data, action and response are insufficient because many of the challenges identified during audit meetings may be considered beyond the scope of the facility to address [17]. Similar challenges have been described in South Africa [18,19].

### Evidence of impact for perinatal mortality audit and review

The 2012 Cochrane review update by Ivers *et al*. concluded that audit and feedback generally could be effective in improving professional practice, although the effects were mostly small to moderate. In the case of a low baseline adherence to recommended practices and more intensive feedback, the relative effectiveness could be greater [2]. A WHO-led meta-review of 110 interventions revealed that audit and feedback was a key facilitator for quality of care improvement [20].

Maternal mortality audit at the population-level have well-proven, sustained benefits across settings though not without challenges [11,21]. A number of high-burden, low-income countries have recently undertaken facility-based maternal death review systems, including Nigeria [22], Malawi [23], Cameroon [24], in Mali and Senegal through the QUARITE trial [25], and in eight countries in sub-Saharan Africa (Burkina Faso, Cameroon, Ethiopia, Mozambique, Nigeria, Uganda) and South Asia (India, Nepal) through FIGO's Leadership in Obstetrics and Gynaecology for Impact and Change (LOGIC) programme [12]. Given that maternal mortality is an increasingly rare event, more facilities are turning to near-miss reviews to develop recommendations to improve care [12,26].

For perinatal mortality specifically, a 2009 systematic review of critical incident audit found no randomised trials, but a meta-analysis of before-and-after effects associated with the introduction of perinatal audits in middle- and low-income countries demonstrated a 30% reduction in mortality [27]. Experience with perinatal audit from high income countries over a number of years has shown that in 30-70% of cases substandard care contributed or caused the death [28]. While limited information is available on the specific attributes of systems which can close the audit loop and reduce perinatal deaths, some evidence of impact is available. In Norway, multidisciplinary perinatal audit has been implemented since 1986 [29]. The perinatal mortality decreased from 13.8 to 7.7 per 1000 live births with better cooperation between hospitals and the implementation of nationwide protocols attributed to the audit process. Nationwide perinatal mortality audits in the Netherlands are the result of a joint effort by government and professional colleges to implement audit in all of the country's 90 obstetric units [30,31]. The MBRRACE-UK (Mothers and Babies Reducing Risk through Audit and Confidential Enquiries across the United Kingdom) has just taken over the long existing confidential enquiries which anonymously investigate maternal deaths, stillbirths and infant deaths. These are supported by the Healthcare Quality Improvement Partnership with funding provided by the four UK Departments of Health plus Eire. In recent years, information on certain congenital anomalies occurring in live births, stillbirths, miscarriages and terminations has also been included. In 2011, a more detailed data collection form was used and a new system for classifying the cause of death was introduced. In Australia while perinatal mortality audit guidelines exist and cause of death review using a single system has been implemented, uptake of substandard care review as part of this process remains haphazard and not reported nationally [32,33]. In New Zealand, maternal and perinatal audit (including in-depth review for substandard care) has been in place since 2006 with all mortality review committees under the auspices of the Health Quality and Safety Commission since 2009 and funded by the national department of health, which follows bi-national guideline recommendations. While the quality and completeness of information for perinatal audit has improved in some regions and improvements in obstetric management have been demonstrated, perinatal audit in high-income countries has a long way to go to ensure mistakes are not repeated and outcomes for mothers and newborns are optimised. More than 35 classification systems for stillbirth causation are currently in use in high-income countries and further research is required into which models work best [28].

**Figure 4:**
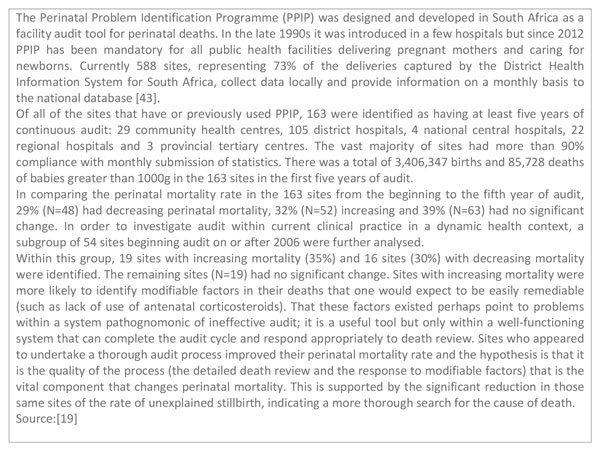
**South Africa's experience with perinatal mortality audit**. PPIP: Perinatal Problem Identification Programme

In low- and middle-income countries there are fewer years of documented experience to draw from overall. The Emergency Obstetric Maternal and Newborn Care six-country cluster randomised control trial included maternal and perinatal audit as one intervention in a package of facility-level quality improvement measures and reported no change in mortality outcomes [34], however, the number of deaths reviewed was used as the metric of successful programming rather than the number of changes instituted following identification through the audit process. According to serial data from South Africa, audit can be a powerful entry point for improved quality of care but only if the identification of deaths and their causes are linked to an analysis of modifiable factors and specific actions (Figure 4) [19].

Implementation of audit programmes is an ongoing process and not a once-off event. In one review, four essential factors were deemed important for audit sustainability including 1) drivers and multidisciplinary teams, 2) clinical outreach visits and supervision, 3) institutional multi-disciplinary review and feedback meetings, and 4) communication and networking between health system levels, facilities and different role-players [18].

### Implementation challenges and evidence-based solutions

Potential challenges to the sustainable implementation of perinatal mortality audit were classified according to the WHO health system building blocks of leadership and governance, health financing, health workforce, essential medical products and technologies, health delivery system, health information system, with community ownership and participation as an additional building block linking the continuum of care (Table 1) [8]. Evidence-based solutions (linked to practice guidelines and existing interventions [35] from the literature) and case studies from different contexts were identified to address each of the challenges.

**Table 1:** Challenges and potential solutions to scaling up perinatal mortality audit by health system building blocks.

Health system building block	Challenges	Potential solutions
Leadership and governance	• Absence of a national policy or strategy on audit • Lack of data collection tools mortality audit meeting guidelines • Lack of prioritisation of audit by policymakers • Culture of blame and fear of potential legal ramifications • Lack of awareness and use of data by government officials • No champions	• National policy with clear implementation plan and decision-tree based on entry-points and system capacity • Standardised tools (paper-based and electronic) available for adaptation at global level • Training for facilitators both integrated into intrapartum care training and stand-alone • Legal protection • High level buy-in for collection and use of data (e.g. from President or Minster of Health)

Health finance	• Lack of funding for audit tool development locally • Training and supervision not currently budgeted • Software and electronic platforms may pose additional financial burden • Opportunity cost of audit committee meetings	• Advocate for inclusion of audit in budget for national and sub-national quality improvement processes • Cost the additional benefit of removing avoidable factors in comparison to extra time spent dealing with missed opportunities

Health workforce	• Overburdened staff do not have time for meetings • Fear of blame, inter-disciplinary mistrust and professional power hierarchies	• Identify champions to lead and participate in the audit committee who will engage not antagonise • Legal protection and confidentiality

Essential medical products and technologies	• Stationery not available for patient records necessary to complete audit • Lack of electronic system means paper-based forms lost, or data not aggregated and shared	• Prioritise stationery procurement • Develop easy to complete patient charts and checklists • More effective records management and retrieval

Health service delivery	• Administration is responsible for many of the necessary changes outside of health worker control	• Ensure facility administrators are members of the audit committee with responsibility to attend meetings periodically if not always • District administrators receive specific, actionable requests from the audit committee

Health information system	• Lack of a centralised database for compiling audit results • No system for notification of perinatal deaths at any level • Poor capacity to use and interpret statistics and create actionable recommendations	• Where practical, consider the use software that generates run chart data, simple graphs, and provides prompts and checklists for addressing recommendations arising from audit

Community ownership and partnership	• Community representatives are rarely engaged in the audit process or informed of the findings • Only facility deaths captured; inequitable representation of true burden of disease and avoidable factors in the community	• Engage a community liaison as a standing member of the audit committee with appropriate confidentiality requirements • Consider community surveillance to inform about perinatal deaths that occur outside the facility and conduct verbal and social autopsy, where feasible

#### Leadership and governance

The lack of a national policy, strategy and/or guidelines for perinatal audit is a limitation in both high-income and low- and middle-income countries, though the availability of a policy alone is not a sufficient measure of success [17]. Even once a policy is enacted, there may not be a process to develop and promote data collection tools, mortality audit meeting guidelines, and clinical criteria by which to audit against. Audit refers to a quality improvement process checked against set standards, whether local, national, or global. Given that few settings have these clinical guidelines formally in place, most perinatal audit processes do not apply formal standards but rather use a team of local experts to determine avoidable factors in each case and identify solutions [11].

Fear of blame - ranging from loss of face amongst peers to potential legal ramifications - has been described as the most significant deterrent to conducting mortality reviews [11]. This lack of audit acceptance is especially pronounced when it is enforced by an external agency [20]. The level of detail that are required to be reported to higher levels (e.g. name of deceased and facility name), may contribute to poorer quality dialogue during mortality reviews, and a shifting of avoidable factors to areas outside of health worker control. A supportive culture at personal, institutional and national level underpinned by the fostering of professionalism and the development of an ethos of safety against a wider supportive and non-punitive environment is needed [11].

Drivers, champions, or "agents of change" have been identified as critical to a sustained programme of audit. These individuals can be managers or health workers, and have been described as "passionate", "committed", "responsible" and "motivated" [18]. These individuals are needed at different levels; ranging from community through to facility as well as management at subnational and national level.

#### Health financing

There is little information in the literature about whether cost is a barrier to implementation of mortality audit systems, though the lack of standard guidelines and tools even in countries with policies mandating audits may reflect that insufficient priority and funding is available for this process. Health records and information management are not prioritised in health budgets though there is increasing donor support for health analytics and information. This is not surprising given that little has been published on the costs of implementing and maintaining electronic health information systems and quality improvement systems generally in low- and middle-income countries [36]. Depending on local capacity, electronic platforms may pose an initial additional financial burden although may save time and money in the longer term [27].

The process of providing orientation and training for audit and software maintenance is low-cost [27]. While the collection of cases and preparing for meetings does reflect a significant input of time from the audit task team, the cost per meeting has been estimated to be around US$200 in two west African settings [37]. The changes required to act on the recommendations from the audit often require a greater outlay of costs, but should result in a more efficient system with targeted health system investments that are more likely to improve quality of care.

#### Health workforce

One challenge identified in the area of health workforce is the tendency to hide behind busy schedules rather than plan and attend audit meetings [18,38]. Integrating audit into routine practice requires formal responsibility for the driver and task team. One way to achieve this is to include audit in job descriptions [18]. Management and teams are also responsible for creating a culture where contributions to review meetings are an integral part of their daily work rather than a strain. Multidisciplinary engagement also needs to be facilitated at higher levels due to potential professional boundaries and conflicts.

There is also a risk of professional power hierarchies dominating the process and silencing less senior voices. To be constructive, the effort requires a team approach and effective facilitation. In Malawi, a quorum was established for review sessions of maternal critical incident audit to take place such that at least one member of all professional cadres had to be present including a member of the district health management team [38].

#### Essential medical products and technologies

Little equipment is required to conduct mortality audit. Primarily, in less-resourced settings the main challenge is that stationery is not available to complete audits [39,40]. In Malawi [23], Tanzania [17] and Uganda [39], the process of documentation has been noted as a barrier to completing audit successfully. However, other than the forms to record case information and record book to keep minuted meeting notes and action item, the patient charts and registers necessary to extract the data may not exist, or may not be adequately designed to capture key information, or, even if they do exist, charts may be missing due to poor storage and management systems [41,42]. Though it is not considered essential equipment, stationery should be prioritised in budgets and designed to be simple, structured, and user-friendly.

#### Health service delivery

Strong health services are those which deliver effective, timely, safe, quality and efficient care. This building block is the main one which audit seeks to strengthen and there are few challenges identified here from the process of audit itself. In fact, delivery of care within the sphere of control of the health worker is the most likely to be impacted by an audit process [19]. However, many of the changes identified through deaths reviews fall outside of health worker control under the purview of administration and management which may demoralise audit teams if recommendations are not followed through and acted upon. Involving facility-level and district management in the local audit team, with a wider national review process affords more accountability at all levels of the health system [23,43].

#### Health information system

A number of critical barriers to perinatal mortality audit implementation were identified linked to the health information system at all stages - in counting deaths, assigning cause of death and avoidable factors, and documenting recommendations and actions taken. In many facilities, and even at regional and national level, there is limited capacity to use and interpret statistics including avoidable factors to create actionable recommendations. Many countries have limited capacity for capturing neonatal deaths, especially those whose births are not registered, and very few countries have any mechanism for tracking stillbirths beyond the facility level. Current systems have selection bias given that perinatal deaths are missed due to lost files or poor recording, and the fact that a large proportion of deaths happen outside the health facility, either at a birth at home, or after discharge [16]. Determining cause of death in the absence of post-mortems can be challenging, particularly for stillbirth. Yet even in better resourced settings, the causes of neonatal death may not be programmatic and linked to obvious solutions [27,44]. Disparate classification systems between CRVS, routine systems, and audit forms may result in duplication and inefficient documentation [45]. Documented avoidable factors may be subjective and depend on the reviewer [46], while the lack of a centralised database for compiling audit results reduces the ability to track trends at all levels of the process. However, the process of death reviews can be used to lead to the production of national standards by level of care, as seen in Uganda [47] and South Africa [48] in order to conduct comprehensive clinical audits against existing criteria, making them less subjective [36,47].

Paper-based systems may appear less costly but may result in lost files, data not being aggregated and shared, and require more people time to manage and collate. Strategies to minimise paper (e.g. cell phone-based audit [39], cloud-storage [49]) have been piloted but not scaled up in many low-income settings. Even in settings where an electronic health information system has been rolled out more widely, like the District Health Information System (DHIS) used in Malawi, Rwanda and South Africa, this may have a limited impact if data quality remains poor [36]. South Africa has demonstrated the benefits of reviewing deaths at facility level while collating data in a centralised database [16,43], but few other countries have managed to do the same.

#### Community ownership and partnership

There are two main ways in which community ownership and partnership can be integrated into a national audit system, both which separate challenges. First, community engagement is necessary for the capture of births and deaths and associated factors at the community level. Capturing events that take place in homes and communities is important in all settings, especially where a significant proportion of women still give birth at home. In audit systems as well as most CRVS, there is only capacity to capture deaths that occur within health facilities. Aside from systems with routine community-level surveillance these systems will not provide a true representation of the burden of disease and avoidable factors in the community. While challenging, involving communities in mortality review does have many potential benefits. In one pilot project in Malawi, community and health facility stakeholders were partnered to identify maternal deaths through verbal autopsy, review causes and associated factors, and take action to prevent further deaths. Community involvement was able to identify additional deaths that may have been otherwise unknown to the health facility. Importantly, the process also resulted in concrete actions at the community and health centre and district hospital level, however the system did not capture information on perinatal deaths [50].

The second aspect of community ownership reflects the need to have communities engaged in the facility-level process of facility-based death review. WHO MDSR technical guidance encourages programme implementers to start in facilities and build capacity for review with health professionals before moving to capturing and reviewing events that take place in communities [1]. For deaths that occur at the facility, community representatives are rarely engaged in the audit process or informed of the findings. Without adequate facilitation and guidance, blame may be transferred to the first delay (decision to seek care) [51], and community-related factors rather than to avoidable factors within the realm of the health provider [48]. When involving the community, either in a one-on-one or group context, facilitators risk alienating the respondents. Engagement with community members must seek to counter the power dynamics and social inequalities in order to get a valid representation of the barriers to seeking and accessing facility-based care [52]. Importantly, the assumption tends to be that patient expectations of death review are low but families can adequately describe the poor care received and know that it can and should be better [52]. Community-related advocacy may benefit from a focus on reducing fatalism surrounding sick newborns as a first step before more effectively engaging the system to demand better services [42].

## Discussion

As an increasing number of countries prioritise and gain experience with mortality audit, more information has been emerging on components for successful audit programmes with implementation viewed as a sustainable and ongoing process and not a once-off event. While key challenges have been identified in each component of the health system, the two main gaps emerged across the literature in the areas of leadership and governance and health information system. There is little debate over whether the task of systematically counting and accounting for deaths is important; the question is how to ensure that data become an instrument to support changes in practice. Audit on its own will not save lives but as part of a package it is a tool for improving quality of care. How can local champions and higher level decision-makers work together to create an accountable system that captures deaths at all levels of the health system with consistent guidelines and training, supportive supervision, with a consolidated central database?

### Getting started

**Figure 5:**
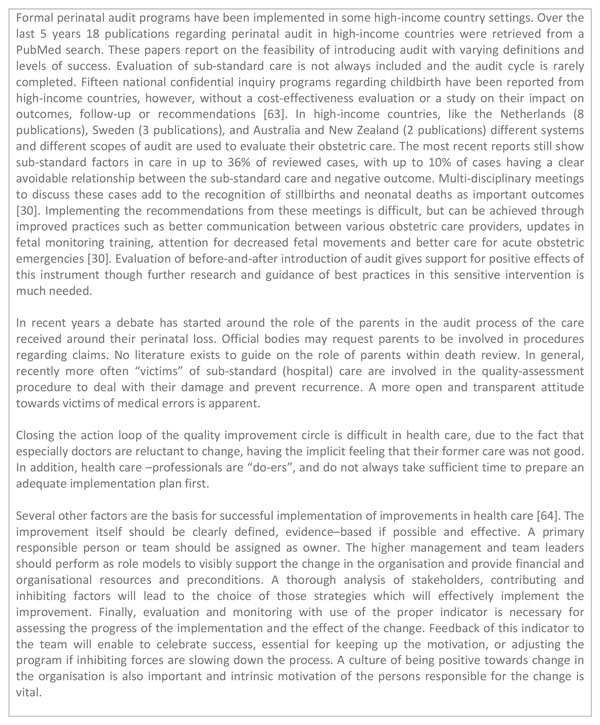
**Learning from perinatal mortality or near-miss audit at scale in high income country settings**.

There are a variety of entry points to introducing perinatal mortality audit. If a decision is taken to introduce audit at national level, there are a number of factors to be considered that applies to both maternal and perinatal audit around the place where deaths will be identified (government facilities, all facilities, community), the scope of implementation (urban areas, sample areas, or full coverage), as well as the depth of the review process (a summary review of a sample of deaths, a summary review of all deaths, or an in-depth review of either a sample or all deaths) [1]. These decisions are often made at the national level and disseminated alongside a national policy and implementation guidelines. Experiences from some high-income countries have shown the potential for sustained, widespread implementation when there is high level national leadership (Figure 5). Where local drivers exist without an overarching national or regional coordinating body, national systems can still arise from the ground-up, as seen in South Africa [27,43].

Even without a national policy or system in place, individual facilities may be encouraged to undertake perinatal mortality audit reviews, linking to existing maternal mortality and morbidity review meetings if they exist. If there are several maternal deaths to review at every meeting teams may consider reviewing at minimum a selection of intrapartum stillbirths and first day neonatal deaths. However, key details should be recorded for each birth and death, including cause of death, even if all cannot be discussed at review meetings.

Ensuring the right stakeholders are on board to prepare for, conduct and participate in audit review meetings is critical. Midwives and obstetricians are in a natural position of leadership given the burden of intrapartum deaths, but first day and later deaths require crossover with other departments and specialities like paediatrics, neonatal nursing, emergency, outpatients, and pharmacy. A steering committee may be established to include representatives of various departments, stakeholders from facility management, as well as the district medical office and community liaison, if applicable. In some settings, the participants may be even further expanded [53]. In the US, multiagency child death review involves coroners, law enforcement, courts, child protective services, as well as health care providers [54] and in England, each local authority has established a multi-disciplinary child death overview panel to review all deaths of children from birth to 18 years in their area [55]. However, such a wide stakeholder group is not essential; there are examples where audit has been successfully initiated and sustained by midwives and community representatives [39].

Leadership, as one of the main challenges identified from the literature, is critical. At national or regional level this includes the overall responsibility for operationalising the audit policy, providing technical assistance for the implementation of audit systems, and monitoring recommendations and follow through. At the local level, it is up to leaders to nurture a conducive culture. Having participants agree to a code of conduct for review meetings, establishing a no-blame environment, and ensuring confidentiality insofar as it's possible contribute to an environment where audit is more likely to be successful [11]. Once a decision is taken to introduce mortality review and a facility-based leadership committee established linking to regional or national systems if they exist, the process of moving through the six-step audit cycle may begin, starting with (1) identifying cases; (2) collecting information; (3) analysing information; (4) recommending solutions; (5) implementing solutions; and (6) evaluating and refining.

### Step 1 - Identifying cases

This may be done from the paper-based or electronic birth or death register. In facilities it also helps to have a lead co-ordinator who checks in with each department for new cases for consideration. Ideally, this should link to CRVS and the routine health information system in addition to providing the basis for the cases for review. The scope of the audit system, including the method of data collection and the outcomes covered depends on local capacity and caseload. This step may be accompanied by a national process to advocate for the introduction or improvement of perinatal death certificates to capture cause of death and maternal condition and link this information to local and national statistics.

### Step 2 - Collecting information

For every death, decisions must be taken as to what information is recorded, where the information is recorded, who records it, and who collates it both for the death review process as well as for reporting to other levels within the system like facility and district level administration, the national ministry of health, as well as inter-sectoral systems such as CRVS. A phased approach--for example, simply capturing the trend of births and deaths, distinguishing between intrapartum stillbirths and intrapartum-related neonatal deaths is a possible first step while gauging the willingness to introduce a maternal and perinatal mortality review, or adding perinatal audit to a more established maternal mortality or near-miss audit. The process of developing a user-friendly form with programmatically relevant causes of death, maternal conditions, and a limited list of avoidable factors clearly linked to recommendations is an essential component of this process. The development of "The WHO application of the International Classification of Disease to perinatal mortality (ICD-PM)" aims to improve the capture of stillbirths and neonatal deaths and link these to contributing maternal conditions in a way that is applicable across all settings. This will assist in standardising and increasing information around the critical time of childbirth.

### Step 3 - Analysing information

**Figure 6:**
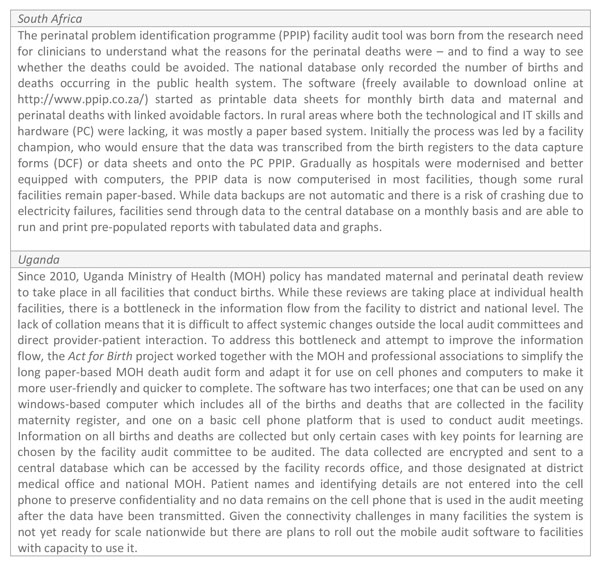
**Using mHealth and technology to facilitate mortality audit**. PPIP: perinatal problem identification programme. PC: personal computer. DCF: data capture forms. MOH: Ministry of Health.

While the perinatal mortality audit process should not primarily be a data producing process, there are minimum analyses and outcomes that should be tallied by the audit committee or designate and presented at scheduled review meetings. These minimum indicators include: the number of normal, assisted and caesarean deliveries; the number of maternal deaths; the number of macerated and fresh stillbirths and early neonatal deaths; and in-facility mortality rates [56]. The number of major complications during labour and delivery and reasons for caesarean section (fetal distress, obstructed labour, failed induction, placental abruptions, post-partum haemorrhage, post-partum infection, severe preeclampsia or eclampsia, etc) may also be collated and presented. If causes of death, maternal conditions, and avoidable factors have been identified, these should be presented, alongside trends, where feasible. Innovation and technology can help particularly in the rapid analysis and presentation of results but shouldn't be the focus of the intervention or a barrier to scale up (Figure 6).

### Steps 4 and 5- Recommend solutions and implement

At the review meeting, the presence of a skilled, independent and accepted chairperson is needed to guide the discussion and refer participants back to best practice guidelines, where available. At this stage, a framework to define what went well and what could have been done differently to provide better care in a no-blame environment can be helpful, along with minuted notes of recommendations, suggested actions and person responsible. While avoidable factors under the purview of administration and management have the capacity to act quickly and this level should not be ignored, it may be more effective to first focus on the avoidable causes within health worker control (e.g. detailed history taking and correct partograph use vs. ambulance availability or lack of resuscitation equipment) and use successes as an advocacy tool to prompt management to further action. In addition to following up on items that have not been completed, it is important to celebrate progress and identify successful changes when they occur.

### Step 6 - Evaluate and refine

Documenting changes over time, through an annual review meeting or report helps identify areas of success and those still needing work. Once the systematic process has begun, maintenance and supervision is critical. A list of questions has been developed to help users assess and reflect on progress at each stage of implementation, from creating awareness of the need for a mortality review process to integrating it into routine practice [18].

### Designing the system for wider scale monitoring and health care improvement

At the national level, a policy, either aligned to maternal mortality review or not, should specifically endorse perinatal mortality audit as a strategy for reducing deaths and improving quality of care. National guidelines for how to set up an audit committee and conduct meetings, clear guidance on information flows, and standardised tools are helpful. National standards to compare against care received may facilitate a more objective assessment of avoidable factors associated with each death. At the local level, this can be done through developing and nurturing champions, particularly advocating for staff designated to oversee the system who are named as part of their job description and able to provide outreach and supervision support to sites as needed. In settings where midwives provide the majority of care at birth and the postnatal period, the system should be developed at a level that midwives can complete the process from start to finish and provide leadership at all levels.

### Way forward

**Figure 7:**
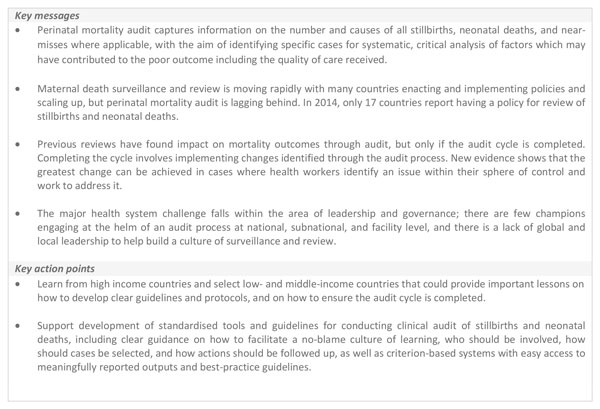
**Key messages and action points**.

The initial country hubs for Every Woman, Every Newborn [8] have experience with perinatal audit, but neither at wide scale. Perinatal mortality audit is a well-known policy in Tanzania but although documentation is widespread the review of these deaths may not be adequately linked to identifying challenges and solutions [17]. Bangladesh was an early adopter of perinatal mortality audit and the government included it as a quality improvement instrument in the national strategy [57] but scale up has been limited with successful review confined to a handful of facilities with dedicated champions. Key messages and action points are summarised in Figure 7.

There is growing demand for information about how to implement and scale up perinatal mortality audit as a central element of a quality improvement strategy; audit came out as the third priority under development domain for the post-2015 research agenda [58]. These outstanding research questions go beyond overarching quality improvement jargon and seek answers to specific, practical implementation questions. Many of the questions about impact, best practices for managing review meetings, and how to follow up on action items in busy maternity units, are also similar to maternal death review and the two should be linked especially where maternal deaths are fewer in number. For many low-income settings, the lack of community participation is also a critical gap and challenge for an equitable process with a positive impact on the families most at risk. There are a number of community participation mechanisms that could be adapted and tested with the aim of building a more comprehensive, effective audit practice. Learning how to scale up the use of perinatal mortality audit was one of the recommendations arising from the Commission on Information and Accountability [59]. The development of WHO guidelines on perinatal mortality audits is expected to help facilitate further testing and expansion of opportunities for research.

## Conclusions

Each death that is reviewed has the potential to tell a story about what could have been done differently to unlock the solutions that should have been available for each woman and baby. Though inputs are needed at every level of the health system and beyond, health workers have the power to change what is in front of them. The system requires leaders to champion the process, especially to ensure a no-fault environment, and to access change agents at other levels to address larger, systemic concerns. It has been suggested that we are entering the third revolution in global public health from metrics and evaluation to accountability and now to improved quality of care [60]. Mortality audit grows out of knowledge of the importance of the first two themes in order to address the third. The benefit of audit and feedback has been acknowledged by development partners and governments to prevent further deaths of mothers, it should also be used to prevent the deaths of their babies.

## List of abbreviations

CARMMA: Campaign for Accelerated Reduction of Maternal Mortality in Africa; CEE/CIS: Central and Eastern Europe and the Commonwealth of Independent States; CRVS: Civil Registration and Vital Statistics; DHIS: District Health Information System; MDSR: Maternal Death Surveillance and Response; Figo: International Federation of Gynecology and Obstetrics; ICD-PM: International Classification of Disease to perinatal mortality; LOGIC: Leadership in Obstetrics and Gynaecology for Impact and Change; MBRRACE-UK: Mothers and Babies Reducing Risk through Audit and Confidential Enquiries across the United Kingdom; WHO: World Health Organization.

## Competing interests

All authors declare they have no competing interests. The assessment of bottlenecks expressed during consultations reflects the perception of the technical experts and may not be national policy. The authors alone are responsible for the views expressed in this article and they do not necessarily represent the decisions, policy or views of the organisations listed, including WHO.

## Authors' contributions

KJK was responsible for the conception, analysis and writing process with oversight from RP, JEL and GL. MM, GL, VF, JJE, TS, PA, AA, EA, NRo, NRh all contributed country data and experiences, wrote sections of text and reviewed drafts of the paper. All named authors contributed to the text and approved the final manuscript.

## Supplementary Material

Additional file 1 Supplementary tables. Click here for file

## Declarations

Publication costs for this supplement were funded by the Bill and Melinda Gates Foundation through a grant to US Fund for UNICEF (Grant ID: OPP1094117), and support from Save the Children's Saving Newborn Lives Programme. Additional funding for the bottleneck analysis was received from USAID (Grant ID: GHA-G-00-07-00007) through UNICEF.

This article has been published as part of *BMC Pregnancy and Childbirth *Volume 15 Supplement 2, 2015: Every Woman, Every Newborn. The full contents of the supplement are available online at http://www.biomedcentral.com/bmcpregnancychildbirth/supplements/15/S2.
