# Supplementary material for: Counting every stillbirth and neonatal death through mortality audit to improve quality of care for every pregnant woman and her baby
Source: BMC Pregnancy Childbirth. 2015 Sep 11;15(Suppl 2):S9. doi: 10.1186/1471-2393-15-S2-S9 (PMC4577789; doi:10.1186/1471-2393-15-S2-S9)
Supplement: Additional file 1 — Supplementary tables. [file 1471-2393-15-S2-S9-S1.docx]

Counting every stillbirth and neonatal death to improve quality of care for every pregnant woman and her baby

Additional file

A. Table S1: Status of maternal death notification policy across Countdown to 2015 countries 2

B. Table S2: Status of perinatal audit policy and systems across Countdown to 2015 countries 8

C. References 11

# A. Table S1: Status of maternal death notification policy across Countdown to 2015 countries

| **Country** | **CD regions** | **2008 Notification of maternal deaths [**[**1**](#_ENREF_1)**]** | **2010 Notification of maternal deaths [**[**2**](#_ENREF_2)**]** | **2012 Notification of maternal deaths [**[**3**](#_ENREF_3)**]** | **2014 Notification of maternal deaths [**[**4**](#_ENREF_4)**]** |
| --- | --- | --- | --- | --- | --- |
| Afghanistan | South Asia | No | No | No | Yes |
| Angola | Eastern/Southern Africa | Yes | Yes | Yes | Yes |
| Azerbaijan | CEE/CIS | Yes | Yes | Yes | Yes |
| Bangladesh | South Asia | Partial | Partial | Yes | No |
| Benin | West/Central Africa | Yes | Yes | Yes | Yes |
| Bolivia | Latin America/Caribbean | - | Partial | Partial | Yes |
| Botswana | Eastern/Southern Africa | - | Yes | Yes | Yes |
| Brazil | Latin America/Caribbean | Yes | Yes | Yes | Yes, but not within 24h |
| Burkina Faso | West/Central Africa | Yes | Yes | Yes | No |
| Burundi | Eastern/Southern Africa | Partial | Partial | Yes | No |
| Cambodia | East Asia/Pacific | Partial | Partial | Yes | Yes, but not within 24h |
| Cameroon | West/Central Africa | No | No | No | Yes, but not within 24h |
| Central African Republic | West/Central Africa | No | No | No | No |
| Chad | West/Central Africa | No | No | Yes | No |
| China | East Asia/Pacific | Yes | Yes | Yes | - |
| Comoros | Eastern/Southern Africa | - | - | - | Yes |
| Congo | West/Central Africa | No | No | No | Yes |
| Congo (DRC) | West/Central Africa | Yes | Yes | Yes | No |
| Côte d'Ivoire | West/Central Africa | Partial | Partial | Yes | Yes |
| Djibouti | Middle East/North Africa | Yes | Yes | Yes | Yes, but not within 24h |
| Egypt | Middle East/North Africa | Yes | Yes | Yes | Yes |
| Equatorial Guinea | West/Central Africa | Partial | Partial | Partial | Yes |
| Eritrea | Eastern/Southern Africa | No | No | No | Yes, but not within 24h |
| Ethiopia | Eastern/Southern Africa | No | No | No | Yes, but not within 24h |
| Gabon | West/Central Africa | No | No | No | Yes |
| Gambia | West/Central Africa | No | No | Yes | Yes |
| Ghana | West/Central Africa | Yes | Yes | Yes | No |
| Guatemala | Latin America/Caribbean | - | Partial | Yes | Yes |
| Guinea | West/Central Africa | Partial | Partial | Partial | No |
| Guinea-Bissau | West/Central Africa | Yes | Yes | Yes | No |
| Haiti | Latin America/Caribbean | No | No | No | No |
| India | South Asia | Yes | Yes | Yes | Yes, but not within 24h |
| Indonesia | East Asia/Pacific | No | No | Yes | Yes |
| Iraq | Middle East/North Africa | Yes | Yes | Yes | Yes |
| Kenya | Eastern/Southern Africa | Yes | Yes | Yes | Yes |
| Korea (DPR) | East Asia/Pacific | - | Partial | Partial | - |
| Kyrgyzstan | CEE/CIS | - | - | Yes | Yes |
| Lao PDR | East Asia/Pacific | - | Partial | Partial | Yes, but not within 24h |
| Lesotho | Eastern/Southern Africa | Yes | Yes | Yes | Yes |
| Liberia | West/Central Africa | Partial | Partial | Yes | Yes |
| Madagascar | Eastern/Southern Africa | Partial | Partial | Yes | No |
| Malawi | Eastern/Southern Africa | Partial | Partial | Yes | Yes |
| Mali | West/Central Africa | Yes | Yes | Yes | No |
| Mauritania | West/Central Africa | Partial | Partial | Partial | No |
| Mexico | Latin America/Caribbean | Yes | Yes | Yes | Yes |
| Morocco | Middle East/North Africa | - | Yes | Yes | Yes |
| Mozambique | Eastern/Southern Africa | Partial | Partial | Partial | Yes, but not within 24h |
| Myanmar | East Asia/Pacific | - | Yes | Yes | Yes, but not within 24h |
| Nepal | South Asia | Partial | Partial | Yes | No |
| Niger | West/Central Africa | No | No | No | No |
| Nigeria | West/Central Africa | No | No | Yes | Yes |
| Pakistan | South Asia | No | No | No | Yes, but not within 24h |
| Papua New Guinea | East Asia/Pacific | - | Yes | Yes | Yes, but not within 24h |
| Peru | Latin America/Caribbean | Yes | Yes | Yes | - |
| Philippines | East Asia/Pacific | - | Partial | Partial | Yes, but not within 24h |
| Rwanda | Eastern/Southern Africa | No | No | No | Yes |
| Sao Tome and Principe | West/Central Africa | - | - | - | Yes |
| Senegal | West/Central Africa | No | No | No | Yes |
| Sierra Leone | West/Central Africa | Partial | Partial | Partial | Yes |
| Solomon Islands | East Asia/Pacific | - | - | Yes | No |
| Somalia | Eastern/Southern Africa | - | No | No | No |
| South Africa | Eastern/Southern Africa | - | Yes | Yes | Yes, but not within 24h |
| South Sudan | Middle East/North Africa | - | - | - | No |
| Sudan | Middle East/North Africa | - | Partial | Partial | Yes, but not within 24h |
| Swaziland | Eastern/Southern Africa | Yes | Yes | Yes | Yes |
| Tajikistan | CEE/CIS | Yes | Yes | Yes | Yes |
| Tanzania | Eastern/Southern Africa | Yes | Yes | Yes | Yes, but not within 24h |
| Togo | West/Central Africa | Yes | Yes | Yes | Yes |
| Turkmenistan | CEE/CIS | No | No | Yes | - |
| Uganda | Eastern/Southern Africa | Partial | Partial | Yes | Yes |
| Uzbekistan | CEE/CIS | - | - | Yes | Yes |
| Viet Nam | East Asia/Pacific | - | - | Yes | No |
| Yemen | Middle East/North Africa | - | No | No | No |
| Zambia | Eastern/Southern Africa | No | Yes | Yes | Yes, but not within 24h |
| Zimbabwe | Eastern/Southern Africa | Yes | Yes | Yes | Yes |
| **Total number of countries** | | **55** | **69** | **73** | **71** |
| **Total Yes responses** | | **22** | **28** | **47** | **51** |

# B. Table S2: Status of perinatal audit policy and systems across Countdown to 2015 countries

|  | **Policy *or* facility-level system for mortality death review** | | **Status of perinatal mortality audit** |
| --- | --- | --- | --- |
| **Country** | **Maternal** | **Stillbirths and neonatal** |  |
| Angola | Yes | Yes | Presidential order which calls for the creation of committees for Maternal and Perinatal Audit. Source: http://www.who.int/woman_child_accountability/countries/AGO_Assessment_Scorecard_draft_web.pdf?ua=1 |
| Azerbaijan | Yes | Yes | Policy includes all facility stillbirths and neonatal deaths to be reviewed. Source: http://www.who.int/maternal_child_adolescent/epidemiology/profiles/maternal/aze.pdf |
| Bangladesh | Yes | Yes | Maternal-Perinatal Death Review (MPDR) based on verbal autopsy without medical certification is in place in selected areas. Online registration of vital events are in process. Source: http://www.who.int/woman_child_accountability/countries/BGD_roadmap_final_web.pdf?ua=1 |
| Gabon | Yes | Yes | Maternal and perinatal death (stillbirths and neonatal deaths) review policy in place since 2010. Source: http://www.who.int/maternal_child_adolescent/epidemiology/profiles/maternal/gab.pdf |
| Gambia | Yes | Yes | Maternal and perinatal death (stillbirths and neonatal deaths) review policy in place since 2010. Source: http://www.who.int/maternal_child_adolescent/epidemiology/profiles/maternal/gmb.pdf |
| Indonesia | Yes | Yes | Ministry of Health published a technical guideline for maternal and perinatal audit. In 2010 there was joint decree that mandated community and health facilities to report all deaths to district civil registration office and cause of death to DHO (eg SMS based reporting) and verbal autopsies for perinatal deaths are taking place in some districts. National capacity to review is available but not institutionalized. Source: http://www.who.int/woman_child_accountability/countries/IDN_roadmap_final_web.pdf |
| Iraq | Yes | Yes* | Profile confirms policy for review of maternal and neonatal deaths (adopted 2010) but this does not include stillbirths. Source: http://www.who.int/maternal_child_adolescent/epidemiology/profiles/maternal/irq.pdf?ua=1 |
| Kenya | Yes | Yes | Neonatal deaths and stillbirths are included in the national Maternal and Perinatal Death Surveillance and Response guidelines and training as of 2014. Source: http://www.who.int/woman_child_accountability/countries/oms-wca-kenya-countryprofils-20140704.pdf?ua=1 |
| Liberia | Yes | Yes* | Policy includes review of maternal and neonatal deaths in facility as of 2010 but stillbirths are not included. Source: http://www.who.int/maternal_child_adolescent/epidemiology/profiles/maternal/lbr.pdf?ua=1 |
| Mexico | Yes | Yes* | Policy includes review of maternal and neonatal deaths in facility before 2000 but not stillbirths, with national panel that meets quarterly to review maternal deaths. http://www.who.int/maternal_child_adolescent/epidemiology/profiles/maternal/mex.pdf?ua=1 |
| South Africa | Yes | Yes | The national perinatal audit system was introduced in the late 1990s in a few hospitals and has been nationally mandated for all public health facilities delivering pregnant mothers and caring for newborns since 2012. Source: http://onlinelibrary.wiley.com/doi/10.1111/1471-0528.12997/abstract |
| Rwanda | Yes | Yes | Neonatal and child death audits started in all district hospitals in 2011. Source: http://www.moh.gov.rw/fileadmin/templates/MOH-Reports/MOH_Booklet_2012_final_September_2013.pdf |
| Tanzania | Yes | Yes | Perinatal deaths included in national Maternal and Perinatal Death Review Guideline since 2006 but are rarely reviewed in practice. Source: http://www.ncbi.nlm.nih.gov/pubmed/25039579 |
| Uganda | Yes | Yes | Perinatal deaths included in national Maternal and Perinatal Death Review Strategy and Guideline since 2010 but guidelines are relatively unknown and not used. Source: http://www.who.int/maternal_child_adolescent/epidemiology/profiles/maternal/uga.pdf?ua=1 |
| Uzbekistan | Yes | Yes | Maternal and neonatal death review policy adopted in 2000 with stillbirths added in 2008. National committee meets quarterly to review. Source: http://www.who.int/maternal_child_adolescent/epidemiology/profiles/maternal/uzb.pdf?ua=1 |
| Zambia | Yes | Yes* | Maternal death and stillbirth review policy adopted prior to 2000. There is no policy for neonatal death review but there is a process for review deaths at facility level. Source: http://www.who.int/maternal_child_adolescent/epidemiology/profiles/maternal/zmb.pdf?ua=1 |
| Zimbabwe | Yes | Yes | Maternal, stillbirth and neonatal death review policy adopted before 2000 for facility and community deaths with a national maternal review panel that meets quarterly. Source: http://www.who.int/maternal_child_adolescent/epidemiology/profiles/maternal/zwe.pdf?ua=1 |

**Notes:**

- All Countdown to 2015 countries were reviewed but only those with maternal mortality audit policies and/or systems were included in this table because there were no countries that had perinatal audit that did not also have maternal mortality audit policies in place.
- Some countries (e.g. India, Brazil) have robust sub-national mortality audit processes in place. Given the lack of ability to systematically review subnational systems, these have not been included in this table.

# C. References

1. Bryce J, Daelmans B, Dwivedi A, Fauveau V, Lawn JE, Mason E, Newby H, Shankar A, Starrs A, Wardlaw T: **Countdown to 2015 for maternal, newborn, and child survival: the 2008 report on tracking coverage of interventions**. *Lancet* 2008, **371**(9620):1247-1258.

2. Bryce J, Requejo JH: **Countdown to 2015 Decade report (2000–2010): Taking stock of maternal, newborn and child survival**. New York: Countdown to 2015 for Maternal, Newborn and Child Health; 2010.

3. Bryce J, Requejo J: **Countdown to 2015 for Maternal, Newborn and Child Health: 2012 report**. In*.* Geneva: World Health Organization; 2012.

4. Mathai M, Dilip TR, Jawad I, Yoshida S: **Strengthening accountability to end preventable maternal deaths**. *Int J Gyn Obst* 2015 (in press).
